# RNA sequencing to determine the contribution of kinase receptor transactivation to G protein coupled receptor signalling in vascular smooth muscle cells

**DOI:** 10.1371/journal.pone.0180842

**Published:** 2017-07-18

**Authors:** Danielle Kamato, Venkata Vijayanand Bhaskarala, Nitin Mantri, Tae Gyu Oh, Dora Ling, Reearna Janke, Wenhua Zheng, Peter J Little, Narin Osman

**Affiliations:** 1 School of Pharmacy, The University of Queensland, Pharmacy Australia Centre of Excellence, Woolloongabba, QLD, Australia; 2 Department of Biotechnology and Environmental Biology, School of Applied Sciences, RMIT University, Bundoora, VIC, Australia; 3 Institute for Molecular Bioscience, The University of Queensland, St Lucia, Qld, Australia; 4 Faculty of Health Sciences, University of Macau, Taipa, China; 5 Xinhua College of Sun Yat-sen University, Tianhe District, Guangzhou, China; 6 Diabetes Complications Group, School of Health and Biomedical Sciences, RMIT University, Bundoora, VIC, Australia; 7 Monash University, Departments of Medicine and Immunology, Central and Eastern Clinical School, Alfred Health, Melbourne, VIC, Australia; Wayne State University, UNITED STATES

## Abstract

G protein coupled receptor (GPCR) signalling covers three major mechanisms. GPCR agonist engagement allows for the G proteins to bind to the receptor leading to a classical downstream signalling cascade. The second mechanism is via the utilization of the β-arrestin signalling molecule and thirdly via transactivation dependent signalling. GPCRs can transactivate protein tyrosine kinase receptors (PTKR) to activate respective downstream signalling intermediates. In the past decade GPCR transactivation dependent signalling was expanded to show transactivation of serine/threonine kinase receptors (S/TKR). Kinase receptor transactivation enormously broadens the GPCR signalling paradigm. This work utilizes next generation RNA-sequencing to study the contribution of transactivation dependent signalling to total protease activated receptor (PAR)-1 signalling. Transactivation, assessed as gene expression, accounted for 50 percent of the total genes regulated by thrombin acting through PAR-1 in human coronary artery smooth muscle cells. GPCR transactivation of PTKRs is approximately equally important as the transactivation of the S/TKR with 209 and 177 genes regulated respectively, via either signalling pathway. This work shows that genome wide studies can provide powerful insights into GPCR mediated signalling pathways.

## Introduction

G protein coupled receptors (GPCRs) are the most prolific and polyfunctional receptors in biology [1, 2]. GPCRs control diverse physiological functions which include, relaxation of blood vessels, acceleration of heart rate, transmission of sight impulses, biorhythms and olfaction [3]. Drugs targeting GPCRs are the largest class of therapeutic agents for diseases including but not limited to: cardiovascular disease, cancer, hypertension and asthma [4–8]. The current paradigm of GPCR signalling covers three major pathways: firstly the well-defined classic signalling pathway which agonist engagement causes G protein binding to the receptor with subsequent downstream signalling leading to functional responses [9–11]. Secondly, the β-arrestin scaffold pathway which leads to the activation of multiple downstream signalling cascades [12]. Two decades ago, the GPCR signalling pathways were complemented by the finding that GPCRs could activate protein tyrosine kinase receptors (PTKRs) leading to the activation of the downstream signalling pathways [13]. This pathway termed transactivation, is the third major signalling pathway of GPCR which was recently expanded to include GPCR mediated transactivation of serine/threonine kinase receptors (S/TKR) [14–18].

The activation of PTKRs by a GPCR immensely expands the range of cellular functions attributable to GPCRs [13, 19, 20]. An example from the vascular biology field is the acute activation of an Angiotensin II receptor in a transactivation independent response leads to a very rapid mobilization of intracellular calcium ions and vascular contraction [21] whereas transactivation of PTKRs leads to a slow vascular remodelling due to cell proliferation and extracellular matrix synthesis [22]. Angiotensin II transactivation goes beyond the vasculature, this signalling pathway has also been shown to be important in kidney [23], heart [24, 25] and cancer [26]. The paradigm of GPCR transactivation signalling has expanded to include the transactivation of the S/TKR and notably the transforming growth factor (TGF)-β receptor (TGFBR1). The phenomenon of transactivation dependent signalling of the PTKR family includes receptors for epidermal growth factor (EGF), platelet derived growth factor, insulin-like growth factor-1 and fibroblast growth factor [13, 27–29]. However GPCR dependent transactivation of the S/TKR is currently restricted to the TGF-β receptor superfamily [15–18, 30–32].

In human vascular smooth muscle cells (VSMCs) the mechanisms involved in PAR-1 mediated transactivation of the PTKR, EGFR, and the S/TKR, TGFBR1, have been partially defined. PAR-1 transactivation of the EGFR involves the activation of cell surface matrix metalloproteinases that cleave and release membrane anchored EGFR ligands. PAR-1 transactivation of the TGFBR1 is mediated by cytoskeletal rearrangement which activates ROCK signalling, leading to the activation of cell surface integrins, which bind to the latent TGF-β complex. This allows for the TGF-β ligand to bind to its receptor. These two transactivation dependent pathways are fully functional in the context that they lead to glycosaminoglycan (GAG) enzyme expression [33] and GAG chain elongation [15]. Beyond this model of proteoglycan synthesis in VSMCs the important transactivation signalling to GPCR signalling is unknown.

To recognize the extent of transactivation dependent signalling a genome-wide study can help identify some promising candidates of PAR-1 transactivation of the TGFBR1 or EGFR. Whole genome RNA sequencing allows for the comprehensive study of the mRNAs in the sample. This new platform has produced high throughput sequencing in the study of various diseases [34–38]. Hannan [39] adapted a similar approach to identify the underlying biology in transactivation of the EGFR. Using a functional siRNA screen of the human genome, the role of 720 kinases was investigated in Angiotensin II mediated transactivation of the EGFR [39]. However in the present study, RNA sequencing analysis was employed to provide an approach to examine the changes induced by PAR-1 transactivation dependent signalling in global gene expression in human coronary artery smooth muscle cells (CASMCs).

## Materials and methods

RNAse-Free DNase set and miRNeasy kit were purchased from Qiagen. Dynabeads® mRNA DIRECT™ Micro kit, Ion PI™ Template OT2 Supplies 200 kit v2, Ion PI™ Template OT2 Reagents 200 Kit v2, Ion PI™ Template OT2 Solutions 200 kit v2, Ion PI™ Sequencing Supplies 200, Ion PI™ Sequencing reagents 200, Ion PI™ Sequencing solutions 200 v2, Ion PI™ Chip preparation solution and Ion Proton™ Chip Adapter where purchased from Life Technologies.

### Cell culture

Human CASMCS (purchased from Lonza Cat No. CC-2583, Lot No. 0000317155) were grown in DMEM (5mM glucose, 10% FBS and 1% antibiotics at 37°C in 5% CO_2_) and were seeded in 60 mm dishes. Cells were grown to confluence then rendered quiescent by serum deprivation for 48 hours. Inhibitors to TGFBR1 (SB431542 10μM) and EGFR (AG1478 5μM) were pre-incubated for 30 mins prior to treatment with thrombin (10 units/ml) for 6 hours. RNA extraction and library preparation followed the manufacturer’s instructions.

### Quality control and read alignment

QuadTrim was used for quality trimming of RNA-sequencing reads. Raw reads with Q-score greater than 20 (>Q20) were retained for further analysis. High quality filtered reads were aligned to the human reference genome (hg19) using Bowtie v2.2.4 and TopHat v2.1.1 [40, 41]. Uniquely mapped reads were then used for counting reads using HTSeq [42].

### Differential gene expression and functional analysis

The genes differentially expressed between the different treatments were identified using edgeR v3.0.7 [43], a Bioconductor package of R statistical environment [44]. This method uses a Poisson distribution to model genic read counts following normalization based on size factors and variance, therefore this software allows normalization of RNA-sequence data based on sequencing depth GC content and gene length for analysis of differentially expressed genes. Differentially expressed genes were defined with log2FC of >0.7 or <-0.7 significant at p value <0.01 (FDR 0.05). STRING (v10.0) (http://string-db.org), a publically available online database of functional interaction [45] was used to identify gene ontology analysis and networks, the differentially expressed genes were analysed. The Venn diagrams were depicted using JVENN an interactive tool to view Venn diagrams and respective lists [46].

### Western blotting

Total cell lysates were resolved on 10% SDS-PAGE and transferred onto PVDF. Membranes were blocked with 5% bovine serum albumin, incubated with ANKRD1 polyclonal antibody (ab88456) and followed by HRP anti-rabbit IgG and ECL detection.

### Assessing mRNA gene expression

The mRNA level of ANKRD1 was determined by real-time polymerase chain reaction (RT-PCR). Total RNA was isolated from treated CASMCs using RNeasy Mini kit (Qiagen) according to the manufactures’ instructions. First strand cDNA was synthesized from 1μg RNA using Quantitect reverse transcriptase kit (Qiagen). Quantitative RT-PCR was performed using Qiagen Rotor Gene Q and QuantiNova SYBR Green PCR Master Mix kit (Qiagen). RT-PCR used human specific ANKRD1 primer sequences. The data was normalized to 8S as the house keeping gene. Relative expression of mRNA levels was quantified using comparative delta delta Ct method.

## Results and discussion

### Gene expression profiles of the treatments

**Fig 1:**
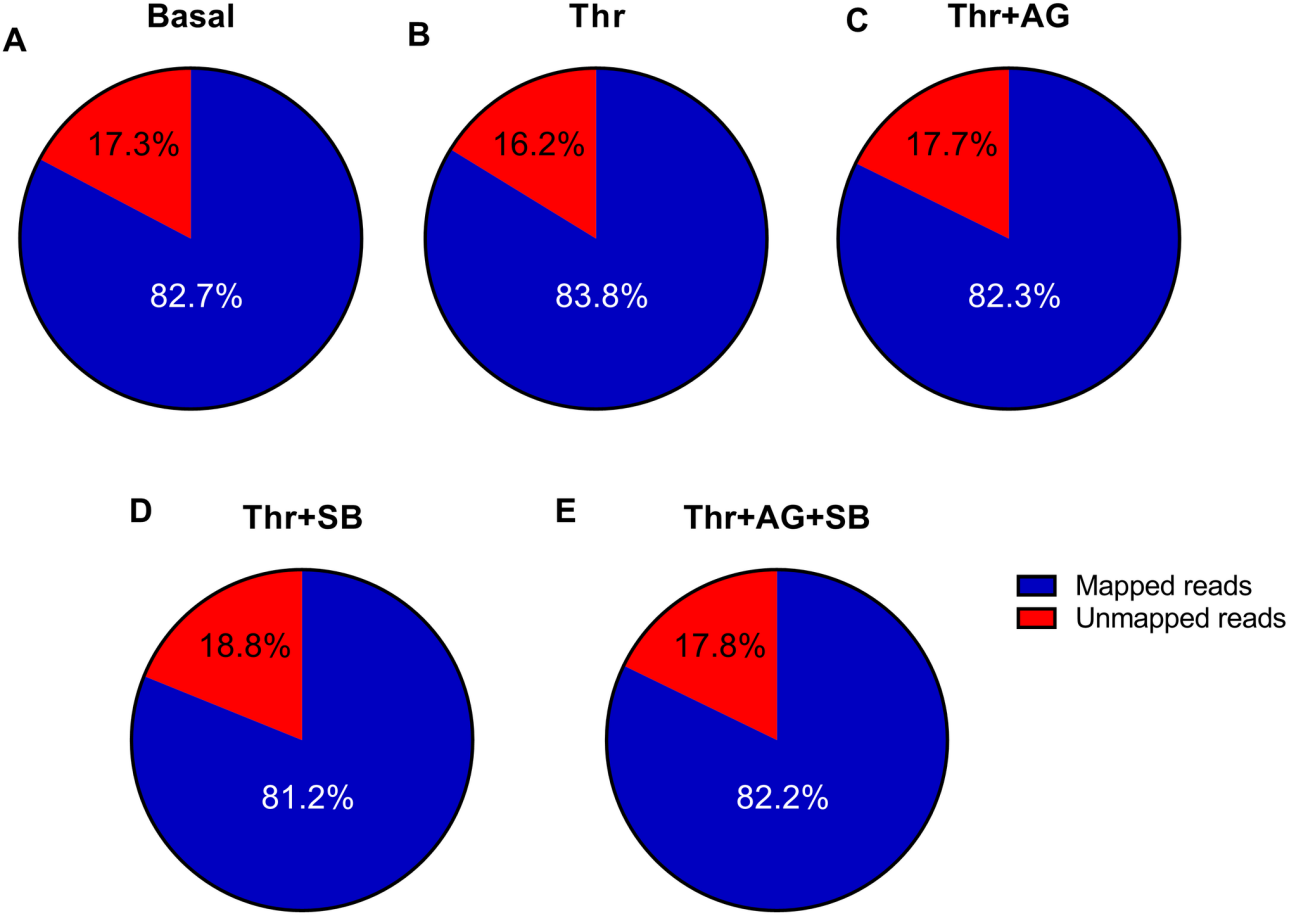
Summary of RNA-sequencing data. Average mapping statistics for each of individual treatments using TopHat. RNA extraction from human CASMCs **A** Untreated **B** treated with thrombin for 6 hours **C** treated with thrombin in the presence of AG1478 (5μM) **D** treated with thrombin in the presence of SB431542 (10μM) **E** treated with thrombin in the presence of both AG1478 and SB431542.

A genome wide RNA-Seq sequencing study was conducted to determine the portion of GPCR signalling which is occurring via transactivation dependent signalling in human CASMCs (Fig 1). The individual treatment groups (Basal, Thrombin, Thrombin+AG1478, Thrombin+SB431542 or Thrombin+AG1478+SB431542) contained 2–4 individual samples pooled together for each of the groups to identify the differentially expressed genes. A total of 3 pooled samples from RNA isolated from untreated CASMCs (Fig 1A), averaged 8 million reads and 82.7% of the total reads were mapped to the human reference genome (hg19). The RNA was harvested from human CASMCs treated with thrombin for 6 hours (Fig 1B), consisted of 4 pooled samples with an average of 12 million reads. This relatively short stimulation period was chosen to focus on primary signalling from GPCR to gene expression, to the exclusion of later responses which may be secondary and beyond responses to the expression of intermediate genes. Among the total number of reads 83.3% of the reads were mapped to the human reference genome (hg19). The differentially expressed genes regulated via PAR-1 mediated transactivation of the EGFR were obtained when CASMCs were treated with thrombin in the presence of EGFR antagonist, AG1478. For this treatment, there was a total of 2 pooled samples (Fig 1C) with an average of 13.5 million reads, 82.3% of these reads were mapped to the human reference genome (hg19). To determine the number of differentially expressed genes occurring via the transactivation of the TGFBR1, CASMCs were treated with thrombin in the presence of TGFBR1 antagonist, SB431542. For this treatment group there was a total of 4 samples pooled together (Fig 1D) with an average read of 13.1 million reads. Among the total number of reads 81.2% of the reads were mapped to the human reference genome (hg19). The treatment showing the genes regulated via dual transactivation dependent signalling pathways were treated with thrombin in the presence of EGFR and TGFBR1 antagonists AG1478 and SB431542, respectively (Fig 1E). A total of 3 individual samples were pooled to give an average of 10.9 million reads. Among the total reads 82.2% of the reads were aligned to the human genome (hg19).

The small sample size in our RNA-Seq study is consistent with other RNA-Seq sequencing studies published which are based on sample sizes of equal to or less than 3 [35]. In human samples greater than 80% of the read pairs can be expected to be mapped to the reference genome [47]. Consistent with the literature [48] the output from our RNA-Seq study shows that over 80% of the reads are mapped to the human genome. This analysis validates that the RNA used to identify the differentially expressed genes are of high quality.

### GPCR mediated transactivation of PTKR and S/TKR accounts for over half of GPCR mediated signalling

**Fig 2:**
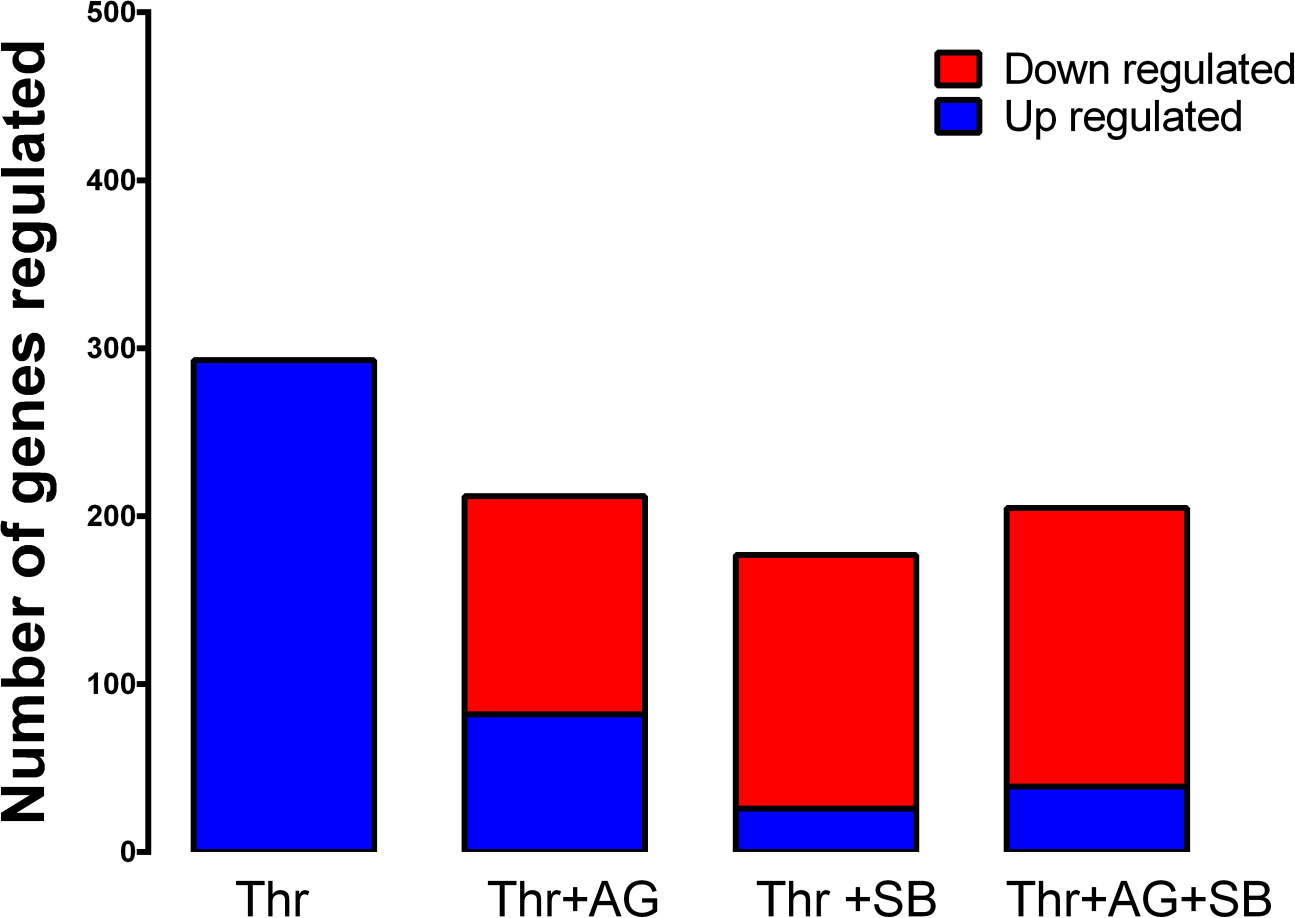
Number of genes regulated by thrombin transactivation of the EGFR and TGBR1 or both in vascular smooth muscle cells. Thrombin compared to basal treatment has a total of 293 genes up regulated. Thrombin transactivation of the EGFR denoted by thrombin in the presence of EGFR antagonist AG1478 has a total of 209 genes differentially regulated (80 up regulated and 129 down regulated). Thrombin transactivation of TGFBR1 denoted by thrombin in the presence of TGFBR1 antagonist SB431542 has a total of 177 genes differentially regulated (26 up regulated and 151 down regulated). Thrombin transactivation of both EGFR and TGFBR1 results in 212 genes differentially regulated (46 up regulated and 166 down regulated).

To assess the contribution of transactivation dependent signalling to overall GPCR signalling, RNA sequencing was conducted. To study the role of PTKR, the EGFR antagonist, AG1478, was used and to study S/TKR transactivation the TGFBR1 antagonist, SB431542, was utilized. We have previously reported that at the concentrations used there is no cross over of inhibitory activity from either drug to either of the target samples [49]. In the present study the differential expression analysis was carried out using a multiple comparison software analysis, edgeR. Each of the treated samples was initially compared to the basal sample. The samples treated with thrombin in the presence of the receptor antagonists were further compared to the CASMCs treated with thrombin alone. The results from this multiple comparison are presented in Fig 2.

Thrombin treatment of human CASMCs resulted in 293 differentially expressed genes increasing in expression as compared to basal. A total of 209 genes were differentially regulated when CASMCs were treated with thrombin in the presence of AG1478. In the presence of AG1478, 80 of the 209 differentially expressed genes had an increase in expression as compared to thrombin treatment alone. The remaining 129 genes differentially regulated by AG1478 had a decrease in expression as compared to thrombin treatment alone (Fig 2). A total of 177 genes are differentially expressed when CASMCs are treated with thrombin in the presence of SB431542. The expression of 26 genes increase in the presence of SB431542 as compared to thrombin treatment alone and 151 genes have a decrease in expression in the presence of the antagonist as compared to thrombin treatment alone (Fig 2). When the two antagonists were combined, a total of 212 genes were differentially expressed, the expression of 166 genes was decreased in the presence of dual antagonists as compared to thrombin and the expression of 46 genes were increased as compared to thrombin treatment alone (Fig 2). Thus these results show that transactivation dependent signalling plays an important role in GPCR signalling with 209 genes regulated via the transactivation of the EGFR, 177 genes regulated via thrombin transactivation of the TGFBR1 and 212 genes regulated via dual transactivation of the EGFR and TGFBR1.

Over the past two decades there have been studies expanding on GPCR mediated transactivation signalling of PTKR specifically the EGFR [39, 50, 51]. GPCR mediated transactivation dependent signalling also spans out to include activation of the TGFBR1. Although this latter pathway was first identified a decade ago [17], there has been a limited amount of studies investigating this transactivation pathway as compared to investigations into the transactivation of the EGFR. Interestingly, the results of this RNA sequencing data show that transactivation dependent signalling via the TGFBR1 is equally as important as transactivation of the EGFR as there is a large number of genes regulated when CASMCs are treated with the respective receptor antagonists in the presence of thrombin.

**Fig 3:**
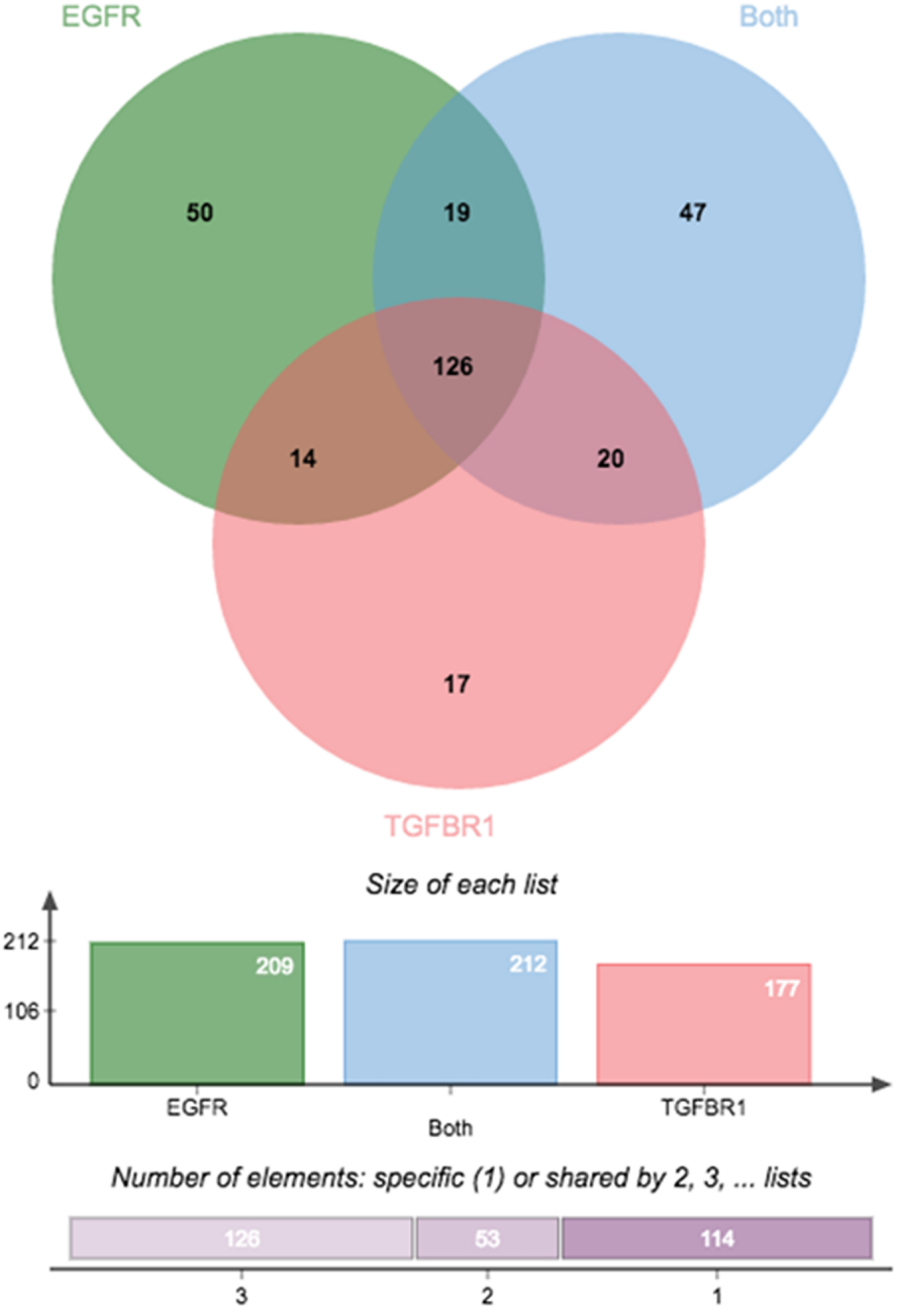
Depicted is the Venn diagram showing the differentially expressed genes of each of the treatments. Thrombin transactivation of the EGFR (209 genes), thrombin transactivation of the TGFBR1 (177 genes) and thrombin transactivation of both EGFR and TGFBR1 (212 genes).

Of the total number of genes differentially regulated by the individual treatments, a Venn diagram was composed to show commonly regulated genes between the individual transactivation dependent pathways (Fig 3). Of the 209 genes differentially expressed by thrombin transactivation of the EGFR, there were 14 genes common to transactivation of TGFBR1 and 19 genes were found to be common in thrombin transactivation of the EGFR and in transactivation of both EGFR and TGFBR1. The genes differentially expressed by thrombin transactivation of the EGFR have 50 genes, which are not common with either of the other two treatments (Fig 3). Thrombin transactivation of the TGFBR1 results in 177 differentially expressed genes, 20 of these genes are in common with thrombin transactivation of both the EGFR and TGFBR1, and 17 genes are specific to transactivation of the TGFBR1. The three individual lists of differentially expressed genes from each of the transactivation pathways share 126 genes which are common. This shows that over 60% of the differentially expressed genes are common between the transactivation dependent signalling pathways. These results indicate that although these two transactivation dependent pathways are independent of each other and their biochemical pathways are mechanistically distinct, they regulate over 60% of the same genes which may indicate that they may be involved in regulating common biological processes.

There are a number of common downstream signalling intermediates which are common to both EGFR and TGFBR1 signalling. MAPKs such as Erk, p38 and Jnk are amongst the common signalling intermediates between the two pathways. MAPKs play a role in transmitting extracellular signals from cell surface receptors to intracellular target and are activated by both TGF-β [52–55] and EGF [56–58]. Similarly other serine/threonine kinases such as PI3K and glycogen synthase kinase 3 are common downstream intermediates to both these signalling pathways [57, 59–63]. Thus common downstream signalling intermediates may allow for convergence; allowing for over 60% of differentially expressed genes to be common between the two pathways.

**Fig 4:**
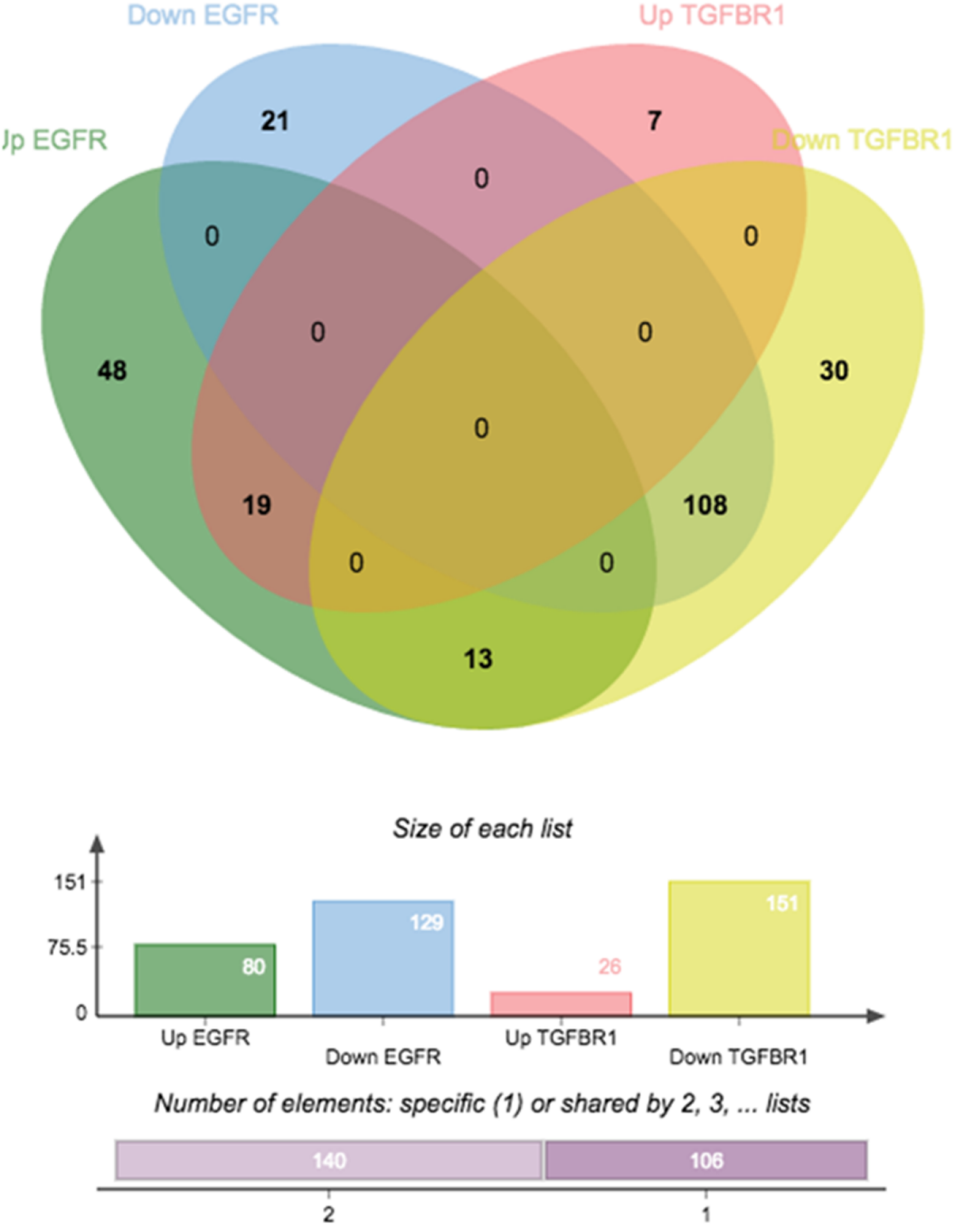
Depicted is the Venn diagram showing the genes up and down regulated by each of the transactivation dependent signalling pathways. 209 genes are differentially expressed via thrombin transactivation of the EGFR (80 upregulated and 129 down regulated) and 177 genes are differentially expressed via thrombin transactivation of the TGFBR1 (26 upregulated and 141 down regulated).

The differential expression data was divided into the up and down regulated genes of each of the transactivation pathways. The Venn diagram in Fig 4 illustrates that from the 80 genes up regulated in thrombin transactivation of EGFR, 19 genes are also commonly up regulated in thrombin transactivation of the TGFBR1. There are 129 genes that are down regulated via thrombin transactivation of the EGFR and of this gene list 108 are commonly down regulated in thrombin mediated transactivation of the TGFBR1. Interestingly there are 13 genes which are up regulated in thrombin transactivation of the EGFR and down regulated in transactivation of the TGFBR1 (Fig 4). These results show that over 80% of the genes down regulated by either pathway are common with only 33% of EGFR mediated transactivation dependent signalling to be specific to EGFR and 20% of genes specific to TGFBR1 transactivation.

### Genes most up and down regulated by the individual transactivation dependent pathways

Overall, there were 209 genes differentially regulated by thrombin transactivation of the EGFR (Fig 2). The top 10 up regulated genes are involved in molecular functions such as cytokine activity which controls cell growth, survival and differentiation as well as genes which are involved in transcription factor activity (Table 1). The top most down regulated genes via EGFR transactivation are involved in receptor protein activity and protein kinase binding (Table 1).

**Table 1:** Genes most up regulated and down regulated by thrombin transactivation of the EGFR in vascular smooth muscle cells.

**Most Up regulated**					
**Gene ID**	**Gene**	**Gene Title**	**Thr**		**Thr+AG**
ENSG00000123358	NR4A1	Nuclear receptor subfamily 4 group A member 1	14.02		19.63
ENSG00000100311	PDGFB	Platelet-Derived Growth Factor Beta Polypeptide	6.74		7.05
ENSG00000109321	AREG	Amphiregulin	6.87		6.92
ENSG00000115009	CCL20	Chemokine (C-C Motif) Ligand 20	4.67		6.42
ENSG00000197632	SERPINB2	Serpin Peptidase Inhibitor, Clade B (Ovalbumin), Member 2	1.34		5.01
ENSG00000136244	IL6	Interleukin 6	3.82		4.63
ENSG00000154319	FAM167A	Family With Sequence Similarity 167, Member A	3.08		4.38
ENSG00000120738	EGR1	Early Growth Response 1	1.39		4.30
ENSG00000117525	F3	Coagulation Factor III (Thromboplastin, Tissue Factor)	2.27		3.83
ENSG00000168389	MFSD2A	Major Facilitator Superfamily Domain Containing 2A	1.41		3.59
**Most down regulated**					
**Gene ID**	**Gene**	**Gene Title**	**Thr**	**Thr+AG**	
ENSG00000127951	FGL2	Fibrinogen-Like 2	16.22	3.12	
ENSG00000153234	NR4A2	Nuclear receptor subfamily 4 group A member 2	9.09	8.15	
ENSG00000102760	RGCC	Regulator Of Cell Cycle	8.99	4.09	
ENSG00000119508	NR4A3	Nuclear receptor subfamily 4 group A member 3	8.11	4.92	
ENSG00000095752	IL11	Interleukin 11	6.14	3.03	
ENSG00000144063	MALL	Mal, T-Cell Differentiation Protein-Like	5.81	1.16	
ENSG00000162494	LRRC38	Leucine Rich Repeat Containing 38	5.48	3.87	
ENSG00000078401	EDN1	Endothelin 1	5.17	1.02	
ENSG00000111859	NEDD9	Neural Precursor Cell Expressed, Developmentally Down-Regulated 9	4.92	2.06	
ENSG00000106278	PTPRZ1	Protein Tyrosine Phosphatase, Receptor-Type, Z Polypeptide 1	4.57	1.03	

There were a total of 177 genes regulated by thrombin transactivation of the TGFBR1 (Table 2). The top 10 most upregulated genes include genes which were associated with GTP-binding including TUBB2A, TUBB4B and TUBA1C (Table 2). Also included in the top most up regulated genes by thrombin transactivation of the TGFBR1 were genes associated with phospholipid binding and phosphatase activity. The majority of the genes down regulated by this transactivation dependent pathway include genes which were associated with transcription factor activity (Table 2).

**Table 2:** Genes most up regulated and down regulated by thrombin transactivation of the TGFBR1 in vascular smooth muscle cells.

**Most Up regulated**					
**Gene ID**		**Gene**	**Gene Title**	**Thr**	**Thr+SB**
ENSG00000171246		NPTX1	Neuronal Pentraxin I	1.62	6.26
ENSG00000197632		SERPINB2	Serpin Peptidase Inhibitor, Clade B (Ovalbumin), Member 2	1.34	4.68
ENSG00000120738		EGR1	Early Growth Response 1	1.39	4.55
ENSG00000168389		MFSD2A	Major Facilitator Superfamily Domain Containing 2A	1.41	3.28
ENSG00000137267		TUBB2A	Tubulin, Beta 2A Class Iia	2.38	3.16
ENSG00000138166		DUSP5	Dual Specificity Phosphatase 5	1.46	3.12
ENSG00000188229		TUBB4B	Tubulin, Beta 4B Class Ivb	1.67	2.90
ENSG00000167553		TUBA1C	Tubulin, Alpha 1c	1.99	2.85
ENSG00000173641		HSPB7	Heat Shock 27kDa Protein Family, Member 7 (Cardiovascular)	1.45	2.66
ENSG00000128228		SDF2L1	Stromal Cell-Derived Factor 2-Like 1	1.41	2.61
**Most down regulated**					
**Gene ID**	**Gene**		**Gene Title**	**Thr**	**Thr+SB**
ENSG00000127951	FGL2		Fibrinogen-Like 2	16.26	4.43
ENSG00000123358	NR4A1		Nuclear receptor subfamily 4 group A member 1	14.07	10.21
ENSG00000153234	NR4A2		Nuclear receptor subfamily 4 group A member 2	9.11	3.93
ENSG00000102760	RGCC		Regulator Of Cell Cycle	9.00	2.59
ENSG00000119508	NR4A3		Nuclear receptor subfamily 4 group A member 3	8.12	3.24
ENSG00000100311	PDGFB		Platelet-Derived Growth Factor Beta Polypeptide	6.76	1.31
ENSG00000095752	IL11		Interleukin 11	6.16	0.84
ENSG00000144063	MALL		Mal, T-Cell Differentiation Protein-Like	5.81	1.84
ENSG00000162494	LRRC38		Leucine Rich Repeat Containing 38	5.50	2.46
ENSG00000078401	EDN1		Endothelin 1	5.18	0.46

Treatment with the two receptor antagonists to assess the contribution of all the transactivation dependent signalling showed 212 genes differentially expressed. The top 10 most upregulated genes of this transactivation pathway were associated with transcription factor activity, cytokine activity and receptor binding activity (Table 3). From the list of differentially expressed genes occurring via the transactivation of both receptors the most down regulated genes are associated with receptor protein and cytokine activity.

**Table 3:** Genes most up regulated and down regulated by thrombin transactivation of the TGFBR1 and EGFR in vascular smooth muscle cells.

**Most Up regulated**				
**Gene ID**	**Gene**	**Gene Title**	**Thr**	**Thr+AG+SB**
ENSG00000123358	NR4A1	Nuclear receptor subfamily 4 group A member 1	14.05	16.91
ENSG00000153234	NR4A2	Nuclear receptor subfamily 4 group A member 2	9.10	20.95
ENSG00000119508	NR4A3	Nuclear receptor subfamily 4 group A member 3	8.12	7.14
ENSG00000115009	CCL20	Chemokine (C-C Motif) Ligand 20	4.69	6.88
ENSG00000136244	IL6	Interleukin 6	3.83	4.50
ENSG00000073756	PTGS2	Prostaglandin-Endoperoxide Synthase 2	3.33	3.81
ENSG00000146374	RSPO3	R-Spondin 3	2.50	2.74
ENSG00000115008	IL1A	Interleukin 1, Alpha	2.45	2.73
ENSG00000143878	RHOB	Ras Homolog Family Member B	2.42	2.70
ENSG00000088826	SMOX	Spermine Oxidase	2.14	2.21
**Most down regulated**				
**Gene ID**	**Gene**	**Gene Title**	**Thr**	**Thr+AG+SB**
ENSG00000127951	FGL2	Fibrinogen-Like 2	16.26	4.14
ENSG00000102760	RGCC	Regulator Of Cell Cycle	9.00	3.48
ENSG00000109321	AREG	Amphiregulin	6.89	3.93
ENSG00000100311	PDGFB	Platelet-Derived Growth Factor Beta Polypeptide	6.75	2.82
ENSG00000095752	IL11	Interleukin 11	6.16	1.51
ENSG00000144063	MALL	Mal, T-Cell Differentiation Protein-Like	5.81	1.65
ENSG00000162494	LRRC38	Leucine Rich Repeat Containing 38	5.51	1.56
ENSG00000078401	EDN1	Endothelin 1	5.19	0.55
ENSG00000111859	NEDD9	Neural Precursor Cell Expressed, Developmentally Down-Regulated 9	4.93	1.61
ENSG00000106278	PTPRZ1	Protein Tyrosine Phosphatase, Receptor-Type, Z Polypeptide 1	4.59	1.23

Interestingly many of the top 10 up and down regulated genes are common between the three treatments. Amongst the top 10 most up regulated genes in thrombin transactivation of the EGFR (Table 1) and via dual transactivation dependent pathways are NR4A1, CCL20 and IL6 (Table 3). These genes are involved in regulating transcription factor, cytokine and growth factor activity. The SERPINB2 and the EGR1 are common in the top up regulated genes in thrombin transactivation of EGFR (Table 1) and TGFBR1 (Table 2). Interestingly although these two genes are in the top upregulated genes of each of the transactivation dependent pathways when the two receptor antagonists were used together these genes did not appear to be in the top 10 most upregulated genes.

In the top most down regulated genes of each of the treatments there were more than 70% commonly expressed genes. The top 10 most down regulated genes dependent on transactivation of the EGFR, TGFBR1 or both pathways have FGL2, PGCC, IL11, MALL, LRRc38 and EDN1 common between the three conditions (Tables 1–3). These genes are commonly associated with cytokine activity and receptor binding. The NR4A2 and NR4A3 genes are common in the top 10 most down regulated genes in thrombin transactivation of TGFBR1 (Table 2) and transactivation of the EGFR (Table 1), interestingly when the two receptor antagonists were applied together (Table 3) these two genes are amongst the top most up regulated genes. The NR4A1 gene found in the most up regulated genes in thrombin transactivation of the EGFR (Table 1) and dual transactivation (Table 3) is amongst the top most down regulated gene via thrombin transactivation of the TGFBR1 (Table 1). Similarly, the PDGFB and the AREG genes associated with protein heterodimerization and EGFR binding, respectively were found in the top 10 most down regulated genes in the dual transactivation dependent signalling (Table 3) and in the top 10 most up regulated genes in thrombin transactivation of the EGFR alone (Table 1). These results show that while genes may be up/down regulated when the dual transactivation pathways are activated, the genes may act opposing in the presence of a single receptor antagonist thus showing the complex nature of transactivation dependent signalling.

In ovarian cancer cells the ET-1 receptor transactivates the EGFR [64, 65] and the vascular endothelial growth factor receptor [66] leading to the expression of EDN1. The expression of EDN1 has also been shown to be involved in colon and prostate cancer cell lines [67, 68]. EDN1 is one of the most down regulated genes in thrombin mediated transactivation of the TGFBR1 (Table 2) and is highly down regulated in the presence of receptor antagonists to both transactivation receptor pathways (Table 3). The role of EDN1 in GPCR mediated transactivation of S/TKR is unknown however EDN1 has a binding site for TGF-β activated Smad transcription factor [69]. In palate development TGF-β treatment increased the expression of EDN1 [70], thus consistent with our results which show that thrombin can transactivate the TGFBR1 which is involved in the regulation of EDN1. Thus showing that thrombin transactivation of the TGFBR1 and EGFR has the potential to be involved in growth related diseases such as cancer and palate development.

### Biological processes regulated by the transactivation dependent pathways

In order to examine the biological processes influenced by each of the transactivation pathways, genes differentially expressed by the different treatments were subjected to functional enrichment analysis based on their gene ontology (GO) annotations. From the 209 differentially expressed and regulated genes by thrombin transactivation of the EGFR, there was 512 GO terms with a false discovery rate (FDR) value of less than 0.05. The top 50 biological terms occurring via thrombin transactivation of the EGFR are listed in Table 4. There was a total of 177 genes differentially regulated by thrombin transactivation of the TGFBR1, from this gene list 428 GO terms with an FDR value of less than 0.05. The top 50 biological terms from the 428 genes associated with thrombin transactivation of the TGFBR1 listed in Table 5. Treatment with two receptor antagonists to account for all the transactivation dependent signalling resulted in 212 differentially expressed genes. From this list of genes 440 terms were enriched by dual transactivation dependent signalling. The top 50 GO terms respective to this signalling cascade are listed in Table 6.

**Table 4:** The top 50 gene ontology terms in the analysis of genes expressed in thrombin transactivation of the EGFR.

GO ID	GO Term	Count	FDR
GO.0051272	positive regulation of cellular component movement	24	3.87E-10
GO.0070887	cellular response to chemical stimulus	57	4.94E-10
GO.0071310	cellular response to organic substance	51	4.94E-10
GO.1901342	regulation of vasculature development	19	4.94E-10
GO.2000147	positive regulation of cell motility	23	4.94E-10
GO.0051094	positive regulation of developmental process	38	1.15E-09
GO.0030335	positive regulation of cell migration	22	1.51E-09
GO.0045765	regulation of angiogenesis	17	6.90E-09
GO.2000145	regulation of cell motility	28	7.31E-09
GO.0030334	regulation of cell migration	27	8.80E-09
GO.1904018	positive regulation of vasculature development	14	8.80E-09
GO.0009653	anatomical structure morphogenesis	51	1.09E-08
GO.0051270	regulation of cellular component movement	29	1.32E-08
GO.0051239	regulation of multicellular organismal process	54	1.85E-08
GO.0042981	regulation of apoptotic process	40	1.89E-08
GO.0043067	regulation of programmed cell death	40	2.16E-08
GO.0001568	blood vessel development	23	2.38E-08
GO.0050793	regulation of developmental process	50	2.41E-08
GO.0022603	regulation of anatomical structure morphogenesis	31	2.59E-08
GO.0042127	regulation of cell proliferation	41	2.59E-08
GO.0001944	vasculature development	23	5.07E-08
GO.0072358	cardiovascular system development	29	7.69E-08
GO.0072359	circulatory system development	29	7.69E-08
GO.0040012	regulation of locomotion	27	9.01E-08
GO.0051240	positive regulation of multicellular organismal process	38	9.01E-08
GO.0010033	response to organic substance	53	1.83E-07
GO.0048514	blood vessel morphogenesis	20	1.88E-07
GO.0051726	regulation of cell cycle	31	1.93E-07
GO.0042221	response to chemical	68	2.21E-07
GO.0048468	cell development	41	2.60E-07
GO.0045766	positive regulation of angiogenesis	12	2.79E-07
GO.0048583	regulation of response to stimulus	63	2.79E-07
GO.0014070	response to organic cyclic compound	28	2.83E-07
GO.0008284	positive regulation of cell proliferation	28	2.92E-07
GO.0071407	cellular response to organic cyclic compound	19	3.23E-07
GO.0071840	cellular component organization or biogenesis	79	5.43E-07
GO.0010941	regulation of cell death	38	7.50E-07
GO.0032879	regulation of localization	49	7.79E-07
GO.0001525	angiogenesis	17	1.04E-06
GO.0051128	regulation of cellular component organization	47	1.04E-06
GO.0009966	regulation of signal transduction	50	1.23E-06
GO.0006928	movement of cell or subcellular component	35	1.73E-06
GO.2000026	regulation of multicellular organismal development	38	1.86E-06
GO.0043065	positive regulation of apoptotic process	22	2.16E-06
GO.0065009	regulation of molecular function	53	2.16E-06
GO.0071495	cellular response to endogenous stimulus	30	2.37E-06
GO.0019220	regulation of phosphate metabolic process	37	3.60E-06
GO.0009605	response to external stimulus	42	3.92E-06
GO.0048519	negative regulation of biological process	70	4.03E-06
GO.1902531	regulation of intracellular signal transduction	35	4.43E-06

**Table 5:** The top 50 gene ontology terms in the analysis of genes expressed in thrombin transactivation of the TGFBR1.

GO ID	GO Term	Count	FDR
GO.0051094	positive regulation of developmental process	34	1.63E-08
GO.0009653	anatomical structure morphogenesis	46	1.66E-08
GO.0030334	regulation of cell migration	25	1.66E-08
GO.0070887	cellular response to chemical stimulus	48	1.66E-08
GO.0071310	cellular response to organic substance	43	1.66E-08
GO.0072358	cardiovascular system development	28	1.66E-08
GO.0072359	circulatory system development	28	1.66E-08
GO.0019220	regulation of phosphate metabolic process	37	8.63E-08
GO.0031399	regulation of protein modification process	38	8.63E-08
GO.0051239	regulation of multicellular organismal process	47	9.10E-08
GO.0048468	cell development	38	1.03E-07
GO.1902531	regulation of intracellular signal transduction	35	1.04E-07
GO.0030335	positive regulation of cell migration	18	1.35E-07
GO.0051270	regulation of cellular component movement	25	1.42E-07
GO.0048519	negative regulation of biological process	65	2.26E-07
GO.0042981	regulation of apoptotic process	34	2.67E-07
GO.0040012	regulation of locomotion	24	2.81E-07
GO.0043067	regulation of programmed cell death	34	2.81E-07
GO.0009888	tissue development	36	3.06E-07
GO.0030154	cell differentiation	53	4.72E-07
GO.0051240	positive regulation of multicellular organismal process	33	4.72E-07
GO.0050793	regulation of developmental process	42	4.79E-07
GO.1901342	regulation of vasculature development	14	5.83E-07
GO.0007507	heart development	19	7.20E-07
GO.0045597	positive regulation of cell differentiation	25	7.20E-07
GO.0009966	regulation of signal transduction	45	7.39E-07
GO.0009892	negative regulation of metabolic process	46	8.03E-07
GO.0065009	regulation of molecular function	48	8.03E-07
GO.0042221	response to chemical	58	1.34E-06
GO.0042325	regulation of phosphorylation	31	1.34E-06
GO.0048583	regulation of response to stimulus	54	1.34E-06
GO.0010033	response to organic substance	45	1.48E-06
GO.0044057	regulation of system process	18	1.60E-06
GO.0051128	regulation of cellular component organization	41	2.63E-06
GO.0051726	regulation of cell cycle	26	3.13E-06
GO.0016477	cell migration	23	3.49E-06
GO.0001932	regulation of protein phosphorylation	29	3.61E-06
GO.0045937	positive regulation of phosphate metabolic process	26	3.67E-06
GO.0001568	blood vessel development	18	3.71E-06
GO.0048869	cellular developmental process	52	4.09E-06
GO.0050790	regulation of catalytic activity	41	4.57E-06
GO.0071407	cellular response to organic cyclic compound	16	4.58E-06
GO.2000026	regulation of multicellular organismal development	33	5.80E-06
GO.0048523	negative regulation of cellular process	58	5.83E-06
GO.0051246	regulation of protein metabolic process	43	6.58E-06
GO.0001944	vasculature development	18	6.70E-06
GO.0042127	regulation of cell proliferation	32	7.24E-06
GO.0010941	regulation of cell death	32	7.49E-06
GO.0014070	response to organic cyclic compound	23	7.63E-06
GO.0006928	movement of cell or subcellular component	30	8.32E-06

**Table 6:** The top 50 gene ontology terms in the analysis of genes expressed in thrombin transactivation of both the EGFR and TGFBR1.

GO ID	GO Term	Count	FDR
GO.0070887	cellular response to chemical stimulus	63	1.11E-12
GO.0071310	cellular response to organic substance	56	1.11E-12
GO.0051094	positive regulation of developmental process	39	9.25E-10
GO.0010033	response to organic substance	58	4.30E-09
GO.0030334	regulation of cell migration	28	4.30E-09
GO.0042221	response to chemical	74	4.30E-09
GO.0048468	cell development	45	1.04E-08
GO.0072358	cardiovascular system development	31	1.08E-08
GO.0072359	circulatory system development	31	1.08E-08
GO.0051240	positive regulation of multicellular organismal process	40	2.15E-08
GO.0042981	regulation of apoptotic process	40	4.01E-08
GO.0051239	regulation of multicellular organismal process	54	4.01E-08
GO.0043067	regulation of programmed cell death	40	4.27E-08
GO.0050793	regulation of developmental process	50	5.47E-08
GO.0030335	positive regulation of cell migration	20	6.37E-08
GO.0051270	regulation of cellular component movement	28	8.18E-08
GO.0071407	cellular response to organic cyclic compound	20	1.08E-07
GO.0071495	cellular response to endogenous stimulus	33	1.14E-07
GO.1901342	regulation of vasculature development	16	1.21E-07
GO.0009653	anatomical structure morphogenesis	49	1.22E-07
GO.0014070	response to organic cyclic compound	29	1.22E-07
GO.0040012	regulation of locomotion	27	1.27E-07
GO.0001568	blood vessel development	22	1.37E-07
GO.0051726	regulation of cell cycle	31	2.93E-07
GO.0048519	negative regulation of biological process	74	3.54E-07
GO.0010941	regulation of cell death	39	4.20E-07
GO.0045597	positive regulation of cell differentiation	28	5.27E-07
GO.0048522	positive regulation of cellular process	74	1.00E-06
GO.0048518	positive regulation of biological process	81	1.01E-06
GO.0042127	regulation of cell proliferation	38	1.22E-06
GO.0051716	cellular response to stimulus	90	1.38E-06
GO.0001944	vasculature development	21	1.41E-06
GO.0060548	negative regulation of cell death	29	1.41E-06
GO.0051241	negative regulation of multicellular organismal process	30	1.45E-06
GO.1901698	response to nitrogen compound	28	1.95E-06
GO.0045765	regulation of angiogenesis	14	1.97E-06
GO.0009892	negative regulation of metabolic process	51	2.92E-06
GO.0048583	regulation of response to stimulus	61	3.06E-06
GO.0032879	regulation of localization	48	3.55E-06
GO.0009888	tissue development	38	3.89E-06
GO.0009890	negative regulation of biosynthetic process	36	4.25E-06
GO.0033993	response to lipid	26	4.25E-06
GO.0043069	negative regulation of programmed cell death	27	4.39E-06
GO.0051128	regulation of cellular component organization	46	4.39E-06
GO.0010629	negative regulation of gene expression	36	4.58E-06
GO.0044057	regulation of system process	19	4.88E-06
GO.0048514	blood vessel morphogenesis	18	5.31E-06
GO.0022603	regulation of anatomical structure morphogenesis	27	5.70E-06
GO.1904018	positive regulation of vasculature development	11	5.71E-06
GO.0010632	regulation of epithelial cell migration	12	5.80E-06

**Fig 5:**
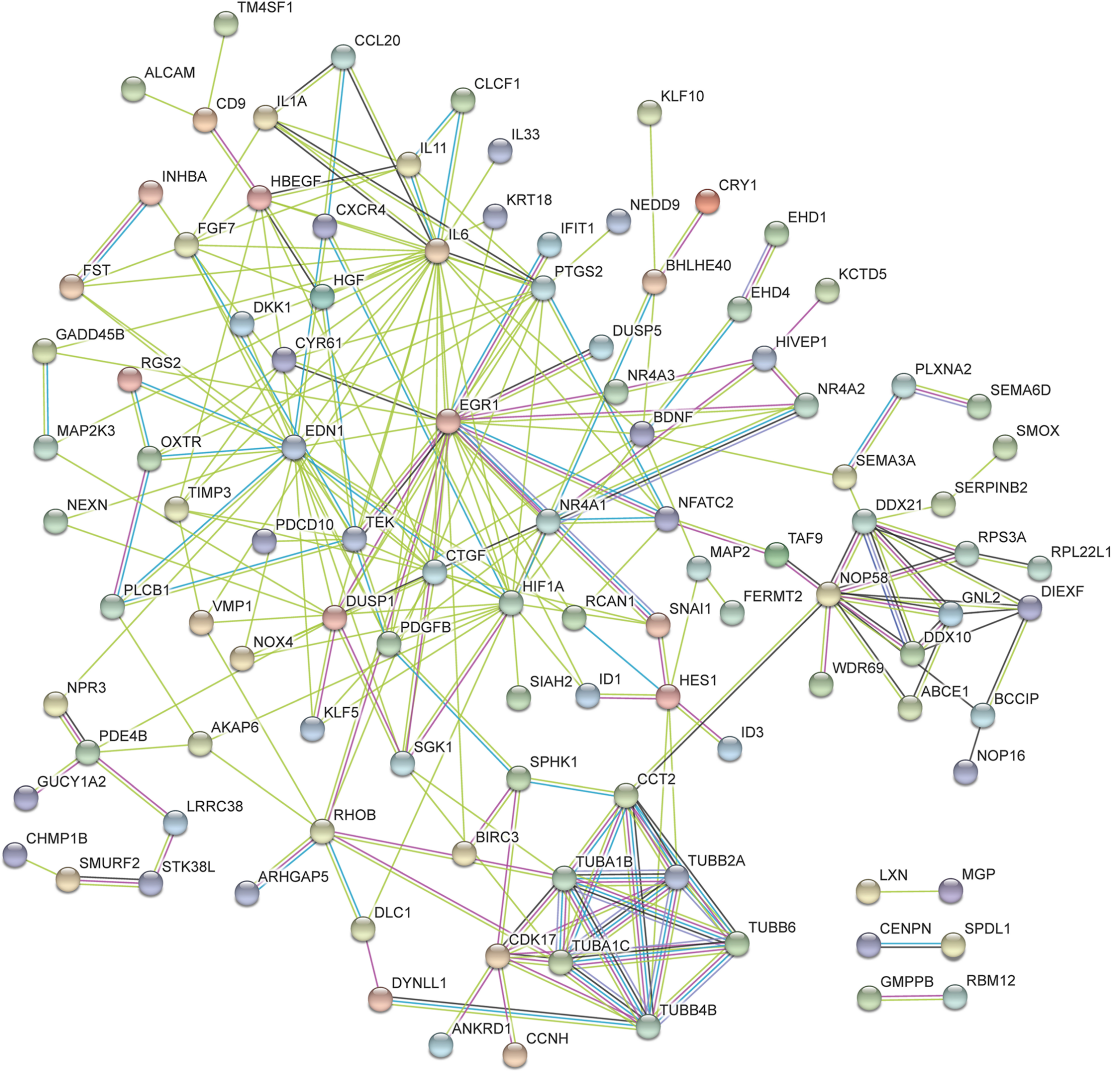
This network represents the differentially expressed genes regulated in thrombin transactivation of the EGFR. The functions of this network include biological processes, molecular functions and cellular component. The color of the nodes indicates the query protein and its first shell of interactions. Protein nodes which are enlarged indicates the availability of 3D protein structure information. The colored lines between the nodes indicated the query protein-protein interactions. The known interactions is a light blue (from curated database) or pink (experimentally determined) lines between the proteins. The predicted interactions use green (gene neighborhood), red (gene fusion) and dark blue (gene co-occurrence) lines. The yellow represents text mining, the black represent co-expression and the purple lines represent protein homology.

**Fig 6:**
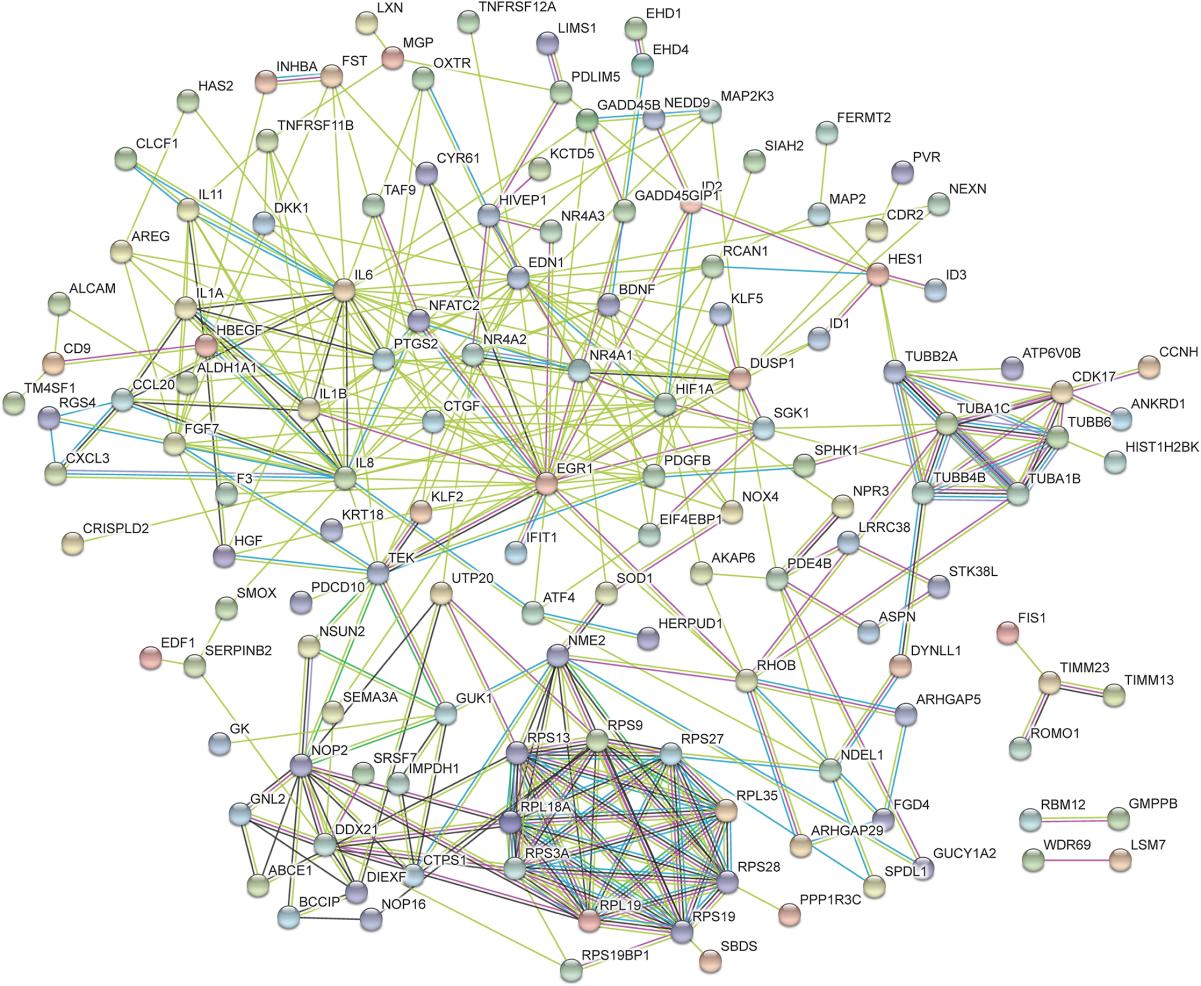
This network represents the differentially expressed genes regulated in thrombin transactivation of the TGFBR1. The functions of this network include biological processes, molecular functions and cellular component. The color of the nodes indicates the query protein and its first shell of interactions. Protein nodes which are enlarged indicates the availability of 3D protein structure information. The colored lines between the nodes indicated the query protein-protein interactions. The known interactions is a light blue (from curated database) or pink (experimentally determined) lines between the proteins. The predicted interactions use green (gene neighborhood), red (gene fusion) and dark blue (gene co-occurrence) lines. The yellow represents text mining, the black represent co-expression and the purple lines represent protein homology.

**Fig 7:**
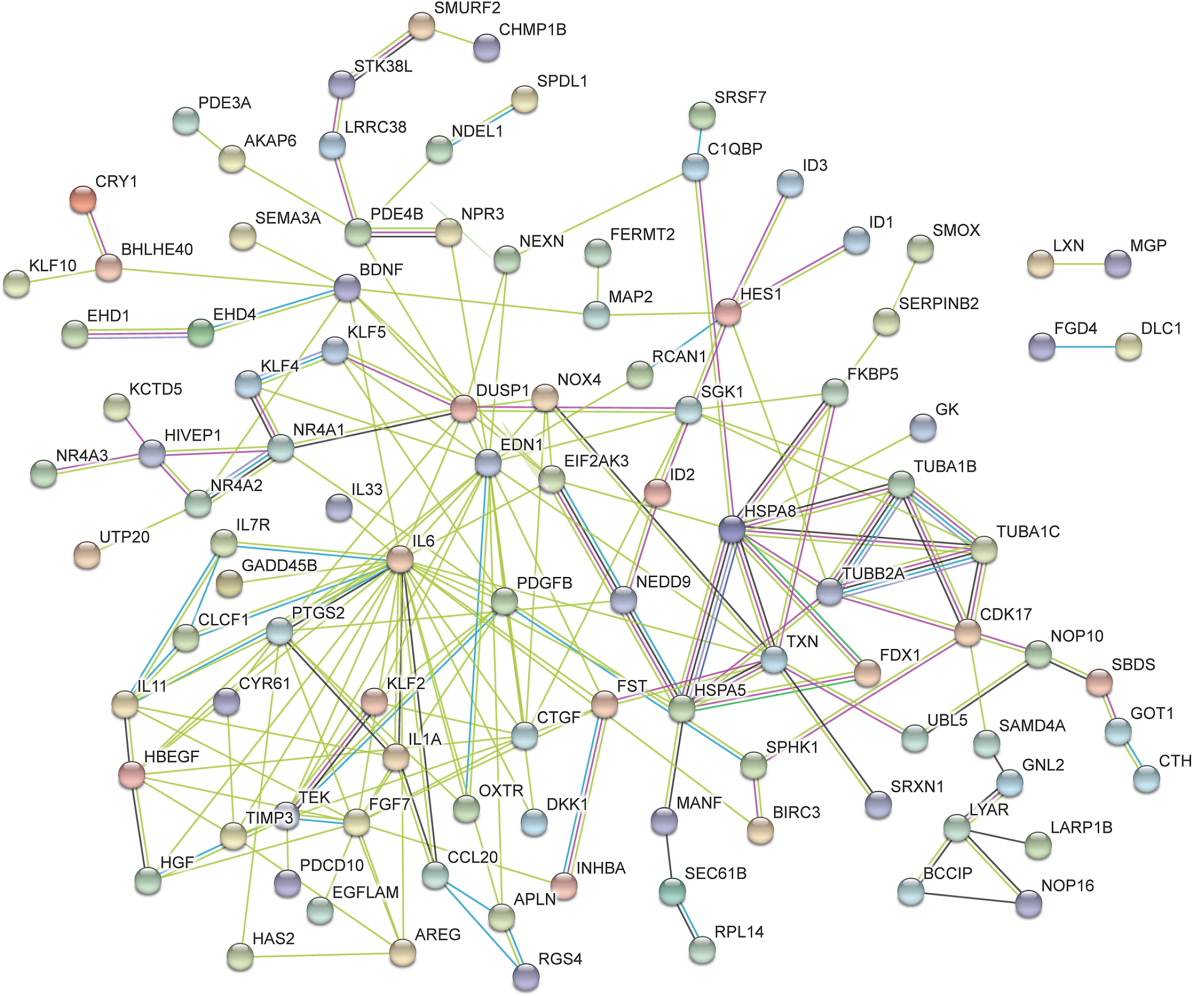
This network represents the differentially expressed genes regulated in thrombin transactivation of both the EGFR and TGFBR1. The functions of this network include biological processes, molecular functions and cellular component. The color of the nodes indicates the query protein and its first shell of interactions. Protein nodes which are enlarged indicates the availability of 3D protein structure information. The colored lines between the nodes indicated the query protein-protein interactions. The known interactions is a light blue (from curated database) or pink (experimentally determined) lines between the proteins. The predicted interactions use green (gene neighborhood), red (gene fusion) and dark blue (gene co-occurrence) lines. The yellow represents text mining, the black represent co-expression and the purple lines represent protein homology.

From the lists of the top 50 processes most significantly enriched by transactivation dependent signalling, 28 terms were found to be common between the three lists (Tables 4–6). Many of these GO terms are associated with regulation of cell activity as well as terms which are associated with vascular development. This includes development of the cardiovascular system, circulatory system, vasculature and blood vessel. From the list of 50 terms associated with dual transactivation dependent signalling (Table 6), five enriched terms were common to thrombin transactivation of the TGFBR1 (Table 5). The common terms include biological processes which are involved in cell growth, cell death and tissue development. Similarly there were five GO terms which were common to dual transactivation dependent signalling (Table 6) and thrombin transactivation of the EGFR (Table 4). Terms common to these two lists include biological processes associated with the regulation of angiogenesis, vasculature development and blood vessel morphogenesis as well as terms involved in cellular activity. When comparing the terms regulated by transactivation of the TGFBR1 (Table 5) and the EGFR (Table 4) there are 5 GO terms which are common to these two pathways which were not found in the top 50 list of terms via dual transactivation (Table 6). These 5 terms are associated with regulation of metabolic processes and signal transduction. Furthermore, network analysis was conducted using String DB, Figs 5–7 show the networks over represented in the gene list of the respective transactivation dependent pathways.

The results above show that thrombin transactivation of the EGFR, TGFBR1 or both receptors is involved in regulating a large number of biological processes 512, 428 and 440 terms, respectively. With the two transactivation dependent pathways being mechanistically distinct [15], the results of these experiments show that over 55% of the top 50 terms regulated are common between the three treatments. Over 60% of the differentially expressed genes described earlier were common to both thrombin mediated transactivation of the TGFBR1 and transactivation of the EGFR. The common differentially expressed genes resulted in over 55% biological terms, which are common between both transactivation pathways. The commonality between the two individual pathways is due the EGFR pathway sharing common downstream signalling intermediates with the TGFBR1 downstream pathway. Serine/threonine kinases such as MAPKs [52–55] are one of many common downstream intermediates to both the transactivation dependent pathways and may play a role in the convergence of the two signalling pathways to regulate the same biological processes.

Our lab has focused on GAG elongation because of its association with atherosclerosis but also because signalling pathways for GAG elongation are distinct from their signalling common responses such as protein signalling and cell proliferation [14–16, 71]. Never the less GAG elongation has also been an excellent response to characterise transactivation dependent signalling and in that context our work has shown how transactivation dependent signalling makes a major contribution to PAR-1 signalling.

### Transactivation dependent signalling is associated with genes regulating vasculature development

To assess the processes influenced by transactivation dependent signalling, the differentially expressed genes were subjected to functional enrichment analysis based on their GO annotations. The biological processes most commonly enriched by the three treatments were linked with vascular development (GO.0072358 Table 7). There were 29 genes associated with cardiovascular system development in thrombin transactivation of the EGFR (Table 4); 28 genes in thrombin transactivation of the TGFBR1 (Table 5) and 31 genes in thrombin transactivation of both the TGFBR1 and EGFR (Table 6). All the genes were up regulated by thrombin when compared to basal and down regulated in the presence of AG1478 with the exception of EGR1, HES1, NR4A1, PDGFB, RHOB, SPHK1 and TIPARP (Table 7). In the presence of thrombin and TGFBR1 antagonist, SB431542, most of the genes associated with cardiovascular system development were down regulated with the exception of EGR1 and TIPARP (Table 7). When looking at dual transactivation dependent signalling associated with cardiovascular system development all genes apart from EGR1, HES1 ID1, KLFS, NR4A1, PTGS2 and RHOB were down regulated as compared to thrombin stimulation alone (Table 7).

**Table 7:** List of genes and respective fold change of expression associated with gene ontology term cardiovascular development. The differentially expressed genes with no fold change presented in the table did not reach the statistical significance cut off (*P<*0.01 and Log2FC>0.7)

Gene ID	Gene Name	Thr	Thr+AG	Thr+SB	Thr+SB+AG
ENSG00000148677	ANKRD1	3.514	0.612	0.962	0.352
ENSG00000183337	BCOR	1.646		0.533	0.543
ENSG00000118523	CTGF	2.662	1.251	0.901	0.933
ENSG00000142871	CYR61	2.631	2.133	2.298	2.024
ENSG00000164741	DLC1	1.755		0.731	0.901
ENSG00000078401	EDN1	5.188	1.013	0.458	0.547
ENSG00000120738	EGR1	1.394	4.249	4.505	3.684
ENSG00000196159	FAT4	1.292		0.601	
ENSG00000176692	FOXC2	1.671			0.694
ENSG00000170961	HAS2	2.047	0.544		0.293
ENSG00000114315	HES1	3.058	3.152	1.154	3.115
ENSG00000100644	HIF1A	1.471	0.639	0.627	
ENSG00000125968	ID1	4.482	0.626	0.775	0.194
ENSG00000115738	ID2	1.302	0.544		0.337
ENSG00000117318	ID3	3.315	1.153	0.814	0.549
ENSG00000121361	KCNJ8	2.129	0.706	0.894	0.637
ENSG00000102554	KLF5	2.679	1.933	2.141	2.690
ENSG00000085276	MECOM	1.673	0.437	0.488	0.486
ENSG00000164134	NAA15	1.639	0.793	0.804	
ENSG00000162614	NEXN	1.815	0.821	0.876	0.886
ENSG00000086991	NOX4	2.015	0.566	0.347	0.423
ENSG00000123358	NR4A1	14.061	19.477	10.102	16.838
ENSG00000180914	OXTR	2.303	0.854	0.616	0.746
ENSG00000138650	PCDH10	1.322	0.667	0.613	0.581
ENSG00000100311	PDGFB	6.773	6.991	1.294	2.835
ENSG00000073756	PTGS2	3.350	3.195	2.066	3.791
ENSG00000127329	PTPRB	1.731			0.829
ENSG00000143878	RHOB	2.416	2.434	1.028	2.691
ENSG00000124216	SNAI1	1.783		0.650	1.174
ENSG00000176170	SPHK1	2.078	2.630	1.731	1.424
ENSG00000120156	TEK	1.936	0.543	0.549	0.576
ENSG00000163659	TIPARP	1.416	2.162	2.084	
ENSG00000006327	TNFRSF12A	1.578		1.309	

The EGR1 (early growth response 1) gene associated with cardiovascular system development was regulated by the three treatments when compared to thrombin (Table 7). EGR1 is involved in the development of atherosclerotic lesions and has been shown to play a role in regulating neointimal thickening in response to vascular injury [72–75]. Thrombin mediated GAG chain elongation evaluated by the mRNA expression of CHST11 and CHSY1 was inhibited in the presence of antagonists to the EGFR and TGFBR1 [33]. However the RNA-Sequencing results show that with treatment of the receptor antagonists in the presence of the thrombin there is an increase in the expression of the EGR1 gene, this may be due to earlier work looking at the pre-inflammatory phase of atherosclerosis. The EGR1 gene is commonly involved in the neointimal thickening which is in the inflammatory phase of atherogenesis.

The ANKRD1 (Ankyrin Repeat Domain 1) gene belongs to the conserved muscle Ankyrin repeat family and is induced in response to inflammation, injury and response to cell stress [76, 77]. The ANKRD1 gene is amongst the differentially expressed genes associated with vascular development. The ANKRD1 gene was involved in the transactivation of the EGFR and the TGFBR1 (Table 7). ANKRD1 is directly mediated via TGF-β mediated signalling as the ANKRD1 promotor region contains a Smad binding motif [78, 79]. The ANKRD1 gene is associated with the actin cytoskeleton signalling network [80], in our model thrombin mediated transactivation of the TGFBR1 activates Rho/ROCK signalling via cytoskeletal rearrangement leading to the activation of cell surface integrins [15]. Thus demonstrating a potential signalling pathway for the activation of the ANKRD1 gene via thrombin transactivation of the TGFBR1. The role of ANKRD1 downstream of the EGFR in unknown, however ANKRD1 is induced by stress activated kinases [81–83] hence we speculate that thrombin mediated transactivation of the EGFR induces the expression of ANKRD1 via the activation of downstream stress activated MAPKs (Erk, p38 and Jnk). MAPK mediated Smad2 linker region phosphorylation correlates with proteoglycan synthesis and GAG chain elongation [84, 85]. In VSMCs thrombin mediated transactivation of the EGFR and TGFBR1 result in the phosphorylation of transcription factor Smad2 in the linker region. Thus GPCR mediated expression of ANKRD1 may signal via Smad2 linker region dependent pathways.

**Fig 8:**
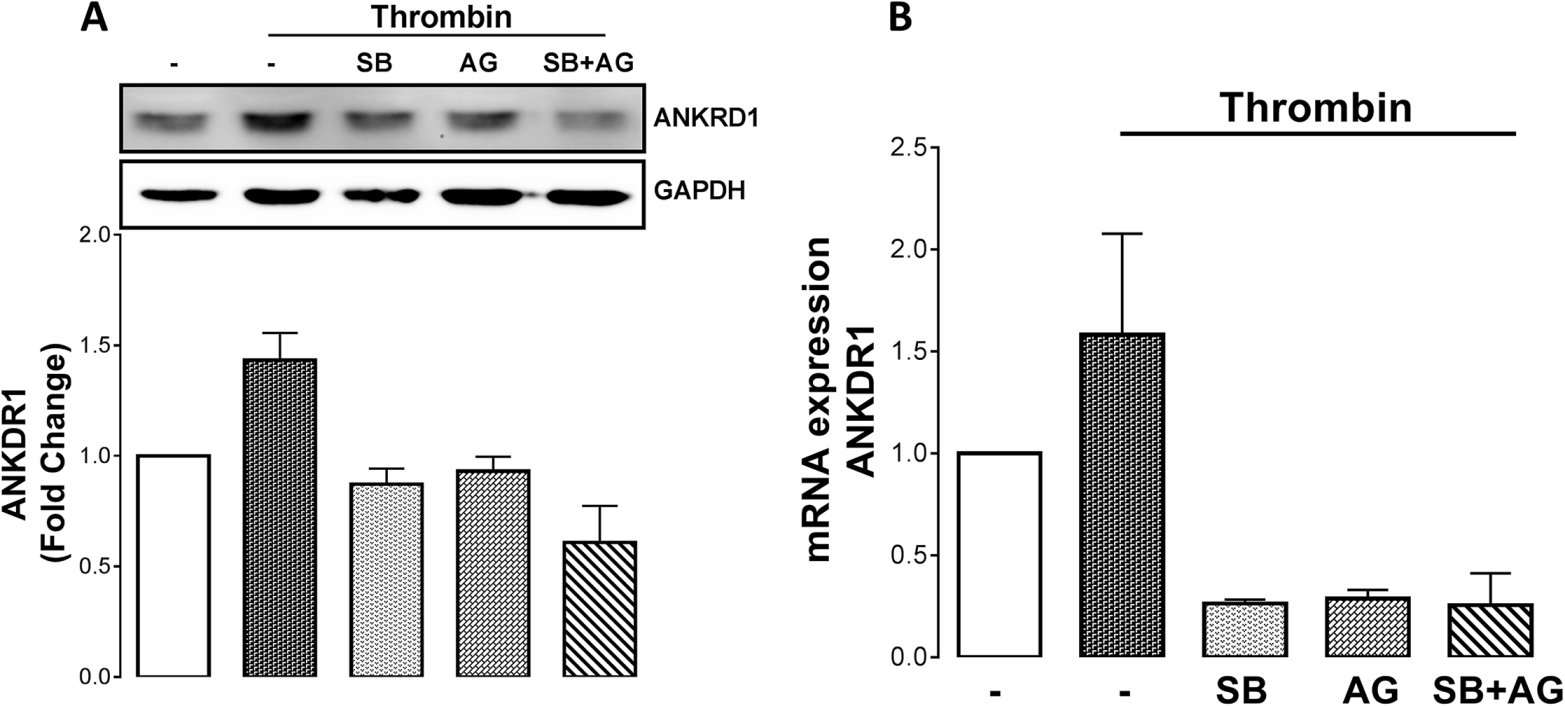
Thrombin mediated ANKRD1 mRNA and protein expression occurs via transactivation of both the EGFR and TGFBR1. CASMCs were pre-treated with AG1478 (5μM), SB431542 (10μM) or both for 30 mins and then exposed to thrombin (10units/ml) for A. 6 hours or B. 1 hour to study the change in ANKRD1 A. mRNA and B. protein expression. The experiments were repeated twice.

To validate the data from the RNA-Seq experiments we investigated thrombin mediated mRNA and protein expression of ANKRD1. Thrombin treated CASMCs lead to a 1.5-fold increase in ANKRD1 mRNA expression (Fig 8A). In the presence of TGFBR1 antagonist, SB431542 and EGFR antagonist, AG1478 individually and together they completely abolished thrombin mediated mRNA expression of ANKRD1 (Fig 8A). The protein expression follows a similar pattern, treatment with thrombin increased ANKDR1 protein levels to 1.5-fold (Fig 8B) in the presence of the two receptor antagonists thrombin mediated ANKDR1 protein expression is completely inhibited. Thus these results validate the genes that were modulated in this RNA-Seq screen.

## Conclusions

GPCRs are one of the most successful class of drug targets on the human genome for the treatment of a variety of diseases [86, 87]. Hence the role of GPCR signalling is of great interest to medical research. GPCRs signal in three main mechanisms, which includes signalling via G proteins to secondary messenger intermediates and via utilization of the signalling pathway and via the utilization of a β-arrestin molecule both which have been extensively reviewed [19, 88]. The third GPCR signalling pathway is via transactivation of PTKR and via transactivation of S/TKR. We subsequently described S/TKR transactivation but again the contribution to overall GPCR signalling was unknown. Hence in this present study, the aim was to determine the contribution of dual transactivation of GPCR mediated signalling by utilizing RNA sequencing to facilitate the identification of the genes regulated by these two transactivation pathways. Using this approach, we have uncovered that thrombin mediated signalling results in 293 differentially expressed genes of these genes approximately 50% of the signalling is occurring via transactivation dependent signalling. Using this approach we have also uncovered that transactivation of the TGFBR1 is as equally important as the transactivation of the EGFR noting that the TGFBR1 transactivation pathway has not yet been as extensively researched.

Hannan et al. [39] used a siRNA approach studied 720 kinase genes associated with Angiotensin II transactivation of the EGFR. Knockdown of 3 genes, TRIO, BMX and CHKA revealed that their activity is required for Angiotensin II mediated transactivation of the EGFR in human mammary epithelial cells [39]. This RNA-Seq sequencing study we explored all the genes that were involved in GPCR mediated transactivation of the EGFR and TGFBR1 in human VSMCs. Although the two transactivation signalling pathways have been reported as to be mechanistically distinct [14, 15] this study shows that these two pathways share in over 65% of the differentially expressed genes. The transactivation dependent signalling pathways shared in over 55% of its top 50 enriched terms, with a majority of these GO terms associated with cardiovascular system development.

The two transactivation dependent signalling pathways share in over 65% of common differentially expressed genes. The most regulated genes of these transactivation mediated pathways are involved in molecular functions such as cytokine activity which controls cell growth survival and differentiation. Cytokine activity is involved in different aspects of cancer, development, treatment and prognosis [89–91]. The EGFR [92–94] and the TGFBR1 [95–97] signalling pathways alone play a role in cancer development. Both the EGFR and TGFBR1 signalling pathways share a common transcription factor, Smad2 linker region. The Smad2 linker region plays a role in cancer development [98–100]. The Smad2 linker region is also associated with other pathophysiological conditions which include but are not limited to proteoglycan synthesis and GAG chain elongation associated with atherosclerosis development [84, 85]. Thus showing that the linker region of the Smad2 may be a transcription factor of interest for to study different biological process occurring via thrombin mediated transactivation of both the EGFR and TGFBR1.

In summary, by generating and utilizing high throughput RNA-Seq data, we have identified that GPCR signalling via transactivation of either of the EGFR or TGFBR1 account for 50% of GPCR signalling. We have identified that these two pathways share in regulation of over 65% of genes of which a majority are involved in the development of the vasculature. This work provides the platform for investigating the molecular basis for transactivation dependent signalling and provides proof that genome wide studies can offer powerful insights into this process.
